# The draft genome of Andean *Rhodopseudomonas* sp. strain AZUL predicts genome plasticity and adaptation to chemical homeostasis

**DOI:** 10.1186/s12866-022-02685-w

**Published:** 2022-12-09

**Authors:** Aisha E. Guardia, Agustín Wagner, Juan P. Busalmen, Cecilia Di Capua, Néstor Cortéz, María V. Beligni

**Affiliations:** 1 Ingeniería de Interfases y Bioprocesos, Instituto de Tecnología de Materiales (INTEMA-CONICET-UNMdP), Mar del Plata, Argentina; 2 Facultad de Ciencias Agrarias, Universidad Nacional de Rosario, Zavalla, Argentina; 3 Facultad de Ciencias Bioquímicas y Farmacéuticas, Instituto de Biología Molecular y Celular de Rosario (IBR-CONICET-UNR), Universidad Nacional de Rosario, Rosario, Argentina; 4 Instituto de Investigaciones Biológicas (IIB-CONICET-UNMdP), Facultad de Ciencias Exactas y Naturales, Universidad Nacional de Mar del Plata, Mar del Plata, Argentina

**Keywords:** Chemical resistance, High-altitude Andean lakes, Pangenomic analysis, Purple non-sulfur bacteria, *Rhodopseudomonas*

## Abstract

The genus *Rhodopseudomonas* comprises purple non-sulfur bacteria with extremely versatile metabolisms. Characterization of several strains revealed that each is a distinct ecotype highly adapted to its specific micro-habitat. Here we present the sequencing, genomic comparison and functional annotation of AZUL, a *Rhodopseudomonas* strain isolated from a high altitude Andean lagoon dominated by extreme conditions and fluctuating levels of chemicals. Average nucleotide identity (ANI) analysis of 39 strains of this genus showed that the genome of AZUL is 96.2% identical to that of strain AAP120, which suggests that they belong to the same species. ANI values also show clear separation at the species level with the rest of the strains, being more closely related to *R. palustris.* Pangenomic analyses revealed that the genus *Rhodopseudomonas* has an open pangenome and that its core genome represents roughly 5 to 12% of the total gene repertoire of the genus. Functional annotation showed that AZUL has genes that participate in conferring genome plasticity and that, in addition to sharing the basal metabolic complexity of the genus, it is also specialized in metal and multidrug resistance and in responding to nutrient limitation. Our results also indicate that AZUL might have evolved to use some of the mechanisms involved in resistance as redox reactions for bioenergetic purposes. Most of those features are shared with strain AAP120, and mainly involve the presence of additional orthologs responsible for the mentioned processes. Altogether, our results suggest that AZUL, one of the few bacteria from its habitat with a sequenced genome, is highly adapted to the extreme and changing conditions that constitute its niche.

**Supplementary Information:**

The online version contains supplementary material available at 10.1186/s12866-022-02685-w.

## Introduction

The genus *Rhodopseudomonas* is composed of gram-negative, purple non-sulfur photosynthetic bacteria from the Alphaproteobacteria class. Members of this genus are widespread in nature, evidenced by the fact that numerous strains have been isolated from very diverse environments [1, 2]. They have extremely versatile metabolisms: they can use carbon dioxide or organic compounds as carbon sources, and light, inorganic or organic compounds as energy sources. They can grow with or without oxygen, fix nitrogen and degrade a great variety of organic compounds [3]. Within this genus, *Rhodopseudomonas palustris* is a model organism for the study of anoxygenic photosynthesis [4, 5]. The sequencing of the first *R. palustris* strain, CGA009, provided the basis for attributing a great deal of the metabolic versatility of this genus to specific genes and pathways [3]. As more strains were sequenced and analyzed phenotypically [1, 2, 6] the remarkable physiological differences between strains became apparent. Comparison of complete genome sequences showed that, although a core set of physiological processes is shared among strains, each behaves as a distinct ecotype that is highly adapted to sediment microenvironments within its natural habitat [2].

In this work, we report the sequencing, genomic comparison and functional annotation of a novel *Rhodopseudomonas* strain, named AZUL, which we previously characterized electrochemically [7]. This strain was isolated from Laguna Azul, an endorheic basin that is part of a number of water bodies collectively known as high-altitude Andean lakes (HAALs), located at the Central Andes region in South America [8]. These habitats experience exposure to severe environmental parameters, including high salinity, osmolarity, UV radiation, barometric pressure, pH and temperature and, as such, are paramount examples of extreme environments [9]. As a consequence, indigenous microbial communities have adapted to combinations of adverse chemical concentrations and physical stress in each particular niche. Several microorganisms isolated from HAALs have been taxonomically identified and characterized, which has shown that the organisms living in these habitats are an important reserve of molecular traits for resistance to environmental conditions such as arsenic and osmotic or UV stress [9–19]. Although metagenomic analyses have begun to tap into the diversity of species within HAALs [20, 21], very few genomes from organisms living in these habitats have been sequenced [8, 9, 22–25].

This work is the first report on the genome sequence of a *Rhodopseudomonas* from HAALs. Our analyses show that strain AZUL features the main characteristics of the genus, reported previously [2, 3] and is also furnished with remarkable specific traits, particularly related to membrane transport, resistance to toxic compounds and responses to nutrient limitation. Altogether, our findings suggest that this strain is highly specialized in maintaining cell homeostasis in a hostile habitat.

## Materials and methods

### Strain and culture conditions

*Rhodopseudomonas* sp. strain AZUL was isolated from a water sample obtained from the high altitude lagoon called Laguna Azul (27°34’ 17.3” S 68°32’ 19.6” W) kindly provided by María Eugenia Farías (PROIMI-CONICET). The water sample was filtered for microorganism enrichment, filters were incubated in Peptone Yeast (PY) medium (1% p/v bactopeptone, 0,05% (w/v) yeast extract, 2 mM MgCl_2_, 2 mM CaCl_2_, 45 μM FeSO_4_) at 28º C under anaerobic illuminated conditions. When brown–red coloration was detected (after 2–3 days), cultures were streaked onto PY-agar plates to select for colonies capable of growing in light, anaerobic conditions.

### DNA isolation, sequencing and genome assembly

*Rhodopseudomonas* sp. AZUL cells were grown in PY medium and harvested by centrifugation at 5,000 × g. Genomic DNA was extracted using the Wizard Genomic DNA Purification kit (Promega). Library preparation, sequencing and data analysis was done at Instituto Nacional de Agrobiotecnología de Rosario (INDEAR) using an Illumina HiSeq 1500 system, according to the manufacturer’s instructions. Briefly, two micrograms of purified DNA were resuspended in 50 µL of TE buffer. DNA fragmentation by nebulization, repair and end-adenylation, adapter ligation, gel purification and enrichment (amplification) were done according to the protocol described in TruSeq® DNA Sample Preparation Guide, Illumina. One microgram of the DNA library was run in a 2100 Bioanalyzer (Agilent Technologies) using the High Sensitivity DNA kit, quantified by qPCR (Light Cycler 480 Roche) using the Kapa Library Quantification kit and normalized to 2 nM. One equimolar pool of the libraries from the same sequencing lane was prepared by mixing 10 µl of each. The pool was used for the generation of clusters in a single lane of the sequencing cell. Paired-end (PE) sequencing was done (2 × 100 bp). Genome assembly starting from paired-end reads was done using the a5pipeline v20140113. Quality of the assembly (completeness, contamination and annotation self-consistency) was estimated using the genome-quality analysis service 'EvalG-EvalCon' within the Genome Annotation service at PATRIC [26]. Preliminary genome annotation was done using the RAST tool kit [27]. The *Rhodopseudomonas* sp. AZUL genome sequence is available at the RAST server (https://rast.nmpdr.org/), using the Login: guest and the Password: guest and also at NCBI GenBank, with the assembly accession GCA_024330085.1.

### Average Nucleotide Identity (ANI) analysis

In addition to AZUL, the genome sequences of 38 *Rhodopseudomonas* strains were retrieved from NCBI genomes (https://www.ncbi.nlm.nih.gov/genome/), all available by January 2022. Genome sequences were compared using OAU, a command line tool for calculating OrthoANI values using the USEARCH algorithm (https://help.ezbiocloud.net/oau-manual/) [28].

### Pangenomic analyses

Genome quality was analyzed using PATRIC [26], similarly to what was previously done with AZUL. Only genomes that had values of completion and consistency higher than 90% and contamination lower than 10% were included in the pangenomic analyses (Additional File 2: Table S1). Clustering and pangenomic analyses were done using the softwares Roary and GET_HOMOLOGUES (GH). GH builds upon orthology-calling approaches based on heuristic pairwise best-match methods, as described [29]. Within the GH package, BLAST results were clustered with the bidirectional best-hit (BDBH) [30], the COGtriangles [31] and the OrthoMCL (OMCL) [32] clustering algorithms. The size of the pangenome was not determined using BDBH since it uses a single reference genome and, thus, cannot track genes not present in the reference. The exponential [33] and binomial mixture models [34] were fitted to the data obtained with OMCL within the GH package to estimate theoretical core and pangenome sizes. The main script get_ homologues.pl was called under default settings, with a minimal sequence identity of 70%. Homologous clusters were computed reporting all clusters (-t) and excluding paralogs. Stringency was added to the analysis by scanning Pfam domain composition of the clusters using hmmscan from HMMER3 package (-D). Exponential decay or binomial mixture models were fitted to the core-genome cluster data by calling the auxiliary script plot_pancore_matrix.pl. For genome composition analysis, the -c flag was used under the exponential model to obtain tables of re-sampled core and pangenome sizes with the same settings as the previous GH analyses. The auxiliary script parse_pangenome_matrix.pl was used to analyze the structure of the pangenome, computing the strict core, relaxed core, shell, and cloud components.

In Roary [35], coding regions from.gff3 files produced by PROKKA [36] were used. The Roary pipeline extracted and converted them to protein sequences, filtered to remove partial sequences and iteratively pre-clustered with CD-HIT [37]. An all-against-all comparison was performed with BLASTP on the reduced sequences with a defined percentage sequence identity of 70%, with all the rest of the parameters set to default. The software then clustered the sequences with MCL and merged them with the pre-clustered results. For pangenomic analysis, the option -s was used in order not to split paralogs into different groups. Phylogenetic reconstruction using the core genome was done using Roary, as part of its default pipeline [35].

### Functional annotation

The output of Roary annotation was used in conjunction with the results of pangenomic analysis to estimate the number of paralogs in protein families, using both default and -s options (splitting and not splitting paralogs). Genes resulting unique or rare in the Roary output were subjected to HMMER clustering analyses for confirmation (using nhmmer) and compared with the remaining strains to determine whether they were absent in other strains or possessed homologs that clustered separately. In addition, the total number of genes from different protein families, classes and subsystems were determined for the 31 strains with the combined outputs of Roary and PATRIC. In the PATRIC website, we used the Metabolomics, Comparative Pathway tool (https://www.patricbrc.org/app/ComparativePathway). Operon visualization was done using Geneious Prime® version 2020.0.5 (https://www.geneious.com/). Further operon editing for Fig. 5 was done using Inkscape 0.92 (https://www.inkscape.org).

### Inductively coupled plasma mass spectrometry (ICP-MS)

The water sample from Laguna Azul was analyzed by Inductively Coupled Plasma Mass Spectrometry (ICP-MS), using the TotalQuant method, for the semi-quantitative estimation of element content. The analysis was done in a Perkin Elmer NexION 350X equipment (CEFOBI- CONICET, Rosario, Argentina) following the manufacturer’s instructions and a single calibration standard containing elements distributed across the mass range, used to create a response table. The complete mass spectrum was determined and interpreted using the Syngistix software. After a total spectrum evaluation for each element, the resulting final isotope intensity counts were summed for each element and were then compared with the stored response table. Each determination was done in duplicates and averaged. With this method, accuracies are within ± 50%.

## Results and discussion

### General features of the *Rhodopseudomonas* sp. AZUL genome

In view of the great adaptability of the *Rhodopseudomonas* genus and the characteristics of the natural habitat of AZUL, we sequenced the genome of this strain. Details on the quality of sequencing and assembly are shown in Additional File 1. Briefly, the genomic library of AZUL was composed of double-stranded DNA fragments of sizes ranging 300–500 bp. Cluster density was 600 K/mm^2^, better than the average value expected for the technology used. The data had good quality parameters, with a 90.8% of all the reads with a Qscore > Q30 (error rate < 0.001, probability of incorrect base call < 1 in 1,000 bases, base call accuracy > 99.9%). Final total reads were 22,300.084, with a 99.14% of the reads passing internal quality filtering procedures (PF reads). The final assembly yielded 120 contigs for a total genome length of 6,050,441 bp, an N50 value of 272,881 and an L50 value of 6. The length of the shortest contig was 304 bp, while the longest was 1,180,838 bp. Quality of the assembly predicted that the genome of AZUL had a 100% completeness and a 2% contamination (Additional File 1).

Table 1 summarizes features of the genome of AZUL in comparison with those of other 38 *Rhodopseudomonas* strains with available genome sequences, as well as information on strain isolation and selected references. The genome of AZUL is larger than 6 Mb, and only *Rhodopseudomonas* sp. AAP120 and *Rhodopseudomonas* sp. BAL398 have genomes of similar size. As a consequence, the number of coding regions in these strains is also larger (Table 1). The GC content is, as for the rest of the strains, at the top end of the spectrum when compared to the genomes of other bacterial groups [38].

**Table 1:** General features of the Rhodopseudomonas genomes analyzed in this study

Proposed name^a^	Genome assembly ID	Size	GC	CDS	Plasmid	Strain isolated from/Isolation information	Reference^b^
*Rhodopseudomonas faecalis JCM 11668T*	* GCA_003217325.1*	*4.07*	*64.20*	*3,681*	*―*	Anaerobic digester treating chicken manure, China	[39]
*Rhodopseudomonas faecalis JSC-3b*	* GCA_000504425.1*	*4.07*	*64.20*	*NA* ^*c*^	*―*	Freshwater canal adjacent to a vegetable field, China	[40]
*Rhodopseudomonas faecalis PSBS*	* GCA_002895035.1*	*3.95*	*64.10*	*NA* ^*c*^	*―*	Swine sewage wastewater, China	[41]
*Rhodopseudomonas palustris 4810*	* GCA_014145305.1*	*5.35*	*63.40*	*4,961*	*―*	Contamination in cultures of Thiospirillum jenense DSM 216^T ^	[42]
*Rhodopseudomonas palustris DSM 123T*	* GCA_900110435.1*	*5.27*	*64.60*	*4,747*	*―*	Surface water or mud	[43]
*Rhodopseudomonas rhenobacensis DSM 12706*	* GCA_014203125.1*	*5.18*	*65.60*	*4,612*	*―*	Sediment of eutrophic pond, Rheinbach, Germany	[44]
*Rhodopseudomonas pentothenatexigens JA575T*	* GCA_900218015.1*	*5.38*	*66.00*	*4,822*	*―*	Paddy soils, India	[45]
*Rhodopseudomonas rutila CGA009*	* GCA_000195775.1*	*5.47*	*64.99*	*4,913*	*1*	--	[3]
*Rhodopseudomonas rutila DSM 126*	* GCA_002937155.1*	*5.39*	*65.00*	*4,895*	*―*	Freshwater pond, Germany: near Zeulenroda	[46]
*Rhodopseudomonas rutila ELI 1980*	* GCA_002026345.1*	*5.65*	*65.09*	*5,090*	*1*	Freshwater pond, Suffolk County, NY, USA.	[47]
*Rhodopseudomonas rutila PS3*	* GCA_003031265.1*	*5.27*	*65.30*	*4,780*	*―*	Paddy field, Taipei City, Taiwan	[48]
*Rhodopseudomonas rutila R1T*	* GCA_003547145.1*	*5.31*	*64.90*	*4,803*	*―*	Rice fields, Japan	[49]
*Rhodopseudomonas rutila TIE-1*	* GCA_000020445.1*	*5.74*	*64.90*	*5,227*	*―*	iron-rich mat, Woods Hole, Massachusetts, USA	[50]
*Rhodopseudomonas rutila YSC3*	* GCA_003031245.1*	*5.37*	*65.20*	*4,871*	*―*	Paddy field, Yilan County, Taiwan	[48]
*Rhodopseudomonas sp. CGMCC 1.2180*	* GCA_013415845.1*	*5.32*	*65.00*	*4,792*	*―*	China	
*Rhodopseudomonas sp. 42OL*	* GCA_001020905.1*	*5.13*	*65.70*	*4,724*	*―*	Sugar refinery waste treatment pond, Castiglion Fiorentino, Italy	[51]
*Rhodopseudomonas sp. AAP120*	* GCA_001295845.1*	*6.16*	*65.70*	*5,516*	*―*	Lagoon, Inner Mongolia, China.	
*Rhodopseudomonas sp. ATH 2.1.18*	* GCA_003591005.1*	*5.63*	*65.40*	*5,084*	*―*	Isolated and purified by C.B. van Niel in 1944 and later transferred to T.E. Meyer at the University of Arizona	
*Rhodopseudomonas sp. ATH 2.1.37*	* GCA_003591275.1*	*5.49*	*65.00*	*4,932*	*―*	Isolated and purified by C.B. van Niel in 1944 and later transferred to T.E. Meyer at the University of Arizona.	[52]
*Rhodopseudomonas sp. B29*	* GCA_000333455.1*	*5.52*	*65.00*	*4,940*	*―*	Rice fields, Japan	
*Rhodopseudomonas sp. BAL398*	* GCA_000935205.1*	*6.12*	*64.20*	*5,844*	*―*	Sea surface water, Baltic Sea	[53]
*Rhodopseudomonas sp. BR0C11*	* GCA_010820945.1*	*5.29*	*65.10*	*4,989*	*―*	Bromeliad Phytotelma, Carite Forest, Puerto Rico	
*Rhodopseudomonas sp. BR0G17*	* GCA_010907025.1*	*5.52*	*65.10*	*5,017*	*―*	Bromeliad Phytotelma, Guajataca Forest, Puerto Rico	
*Rhodopseudomonas sp. BR0M22*	* GCA_010907035.1*	*5.29*	*65.20*	*4,761*	*―*	Bromeliad Phytotelma, Maricao Forest, Puerto Rico	
*Rhodopseudomonas sp. Cfx3-05*	* GCA_013377015.1*	*4.83*	*64.40*	*4,511*	*―*	Metagenomic assembly from the Ca. Chlorohelix allophototropha enrichment, Canada: near Kenora	
*Rhodopseudomonas sp. GJ-22*	* GCA_007005445.1*	*5.04*	*65.80*	*4,550*	*―*	Wastewater from a pesticide factory, Changsha (Hunan, China)	[54]
*Rhodopseudomonas sp. HaA2*	* GCA_000013365.1*	*5.33*	*66.00*	*4,731*	*―*	Uncontaminated freshwater marsh sediment location A, Haren, The Netherlands	
*Rhodopseudomonas sp. NC 1818*	* GCA_016215605.1*	*5.79*	*66.20*	*5,274*	*―*	Metagenomic assembly obtained from the groundwater metagenome BioSample: SAMN15459608	[55]
*Rhodopseudomonas sp. RCB100*	* GCA_016584445.1*	*5.46*	*64.99*	*4,907*	*1*	Creek soil, Cascadilla Creek, Ithaca, NY, USA	[56]
*Rhodopseudomonas sp. RI 341*	* GCA_016124795.1*	*5.14*	*63.00*	*4,784*	*―*	Hot springs and caldera lake in Raoul Island, New Zealand.	
*Rhodopseudomonas sp. SK50-23*	* GCA_018279705.1*	*5.86*	*61.90*	*5,281*	*―*	Nonpolluted garden soil, Japan	[57]
*Rhodopseudomonas sp. WA056*	* GCA_010906995.1*	*5.06*	*65.80*	*4,562*	*―*	Water reservoir, Puerto Rico	
*Rhodopseudomonas sp. XCP*	* GCA_003226555.1*	*5.59*	*65.20*	*5,096*	*―*	Contaminant of a green bacterial culture, La Jolla, CA, USA	[58]
*Rhodopseudomonas thermotolerans JA576*	* GCA_003387125.1*	*5.38*	*66.00*	*4,825*	*―*	Paddy soils, India	[45]
*Rhodopseudomonas palustris BisB5*	* GCA_000013685.1*	*4.89*	*64.40*	*4,397*	*―*	Uncontaminated freshwater marsh sediment location B, Haren, The Netherlands	[1]
*Rhodopseudomonas sp. BisB18*	*PRJNA15750* ^*c*^	*5.51*	*65.00*	*4,867*	*―*	Uncontaminated freshwater marsh sediment location B, Haren, The Netherlands	[1]
*Rhodopseudomonas sp. BisA53*	*PRJNA15751* ^*c*^	*5.51*	*64.40*	*4,852*	*―*	Uncontaminated freshwater marsh sediment location A, Haren, The Netherlands	[1]
*Rhodopseudomonas sp. DX-1*	* GCA_000177255.1*	*5.4*	*65.40*	*4,849*	*―*	Microbial fuel cell	[59]
*Rhodopseudomonas sp. AZUL*	* GCA_024330085.1*	*6.05*	*65.60*	*5,596*	*―*	High altitude shallow lake, Andes Region, Catamarca, Argentina	[7], this work

^a^Taxonomy as proposed by Imhoff et al. [60].

^b^Main reference cited when available

^c^Not available

**Fig. 1:**
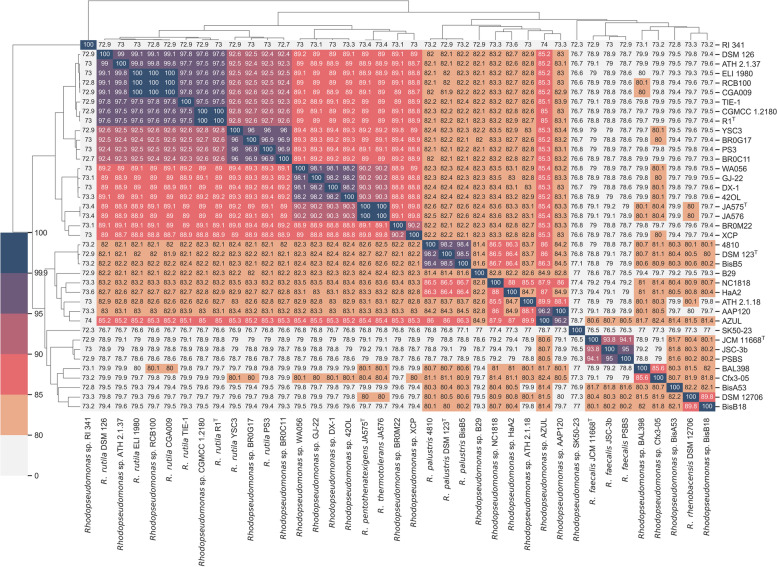
Average Nucleotide Identity (ANI) values of strains within the *Rhodopseudomonas* genus. Thirty-nine strains were analyzed using the OAU software. The percentages of identity are highlighted as a color scale shown to the right of the image. A similarity tree is shown on top of the identity matrix. The name of each strain follows the taxonomy proposed in Imhoff et al. [60]

In order to establish the similarity of AZUL with other strains, we compared the ANI values of the 39 *Rhodopseudomonas* strains in Table 1. Figure 1 shows that the genome sequence of AZUL is most similar to that of *Rhodopseudomonas* sp. strain AAP120 and, by the high ANI value (96.2%), it could be presumed that both strains belong to the same species. The cluster formed by AZUL and AAP1120 lies within a larger group that contains the *Rhodopseudomonas* sp. strains ATH 2.1.18 (ANI 89.9%), HaA2 (ANI 87%), NC1818 (ANI 87.9%), B29 (ANI 84.9%), BisB5 (ANI 86.3%) and two *Rhodopseudomonas palustris* strains, *R. palustris* strain 4810 (ANI 86%) and the type strain *R. palustris* DSM 123^ T^ (ANI 86%). As previously determined for AAP120 [60], these strains could be recognized as separate species compared to AZUL and AAP120 (ANI values lower than 90%).

Figure 1 also shows that the genome sequence of AZUL is quite different from those of the *R. pentothenatexigens* type strain JA575^T^ and several *R. rutila* strains, including the type strain R1^T^. The ANI values between AZUL and these strains are approximately 85%, which indicates a clear separation at the species level. The ANI values between AZUL and several *R. faecalis* strains, including the type strain JCM 11668^ T^, are even lower and range 80%.

### *Rhodopseudomonas* pangenomic analysis

**Fig. 2:**
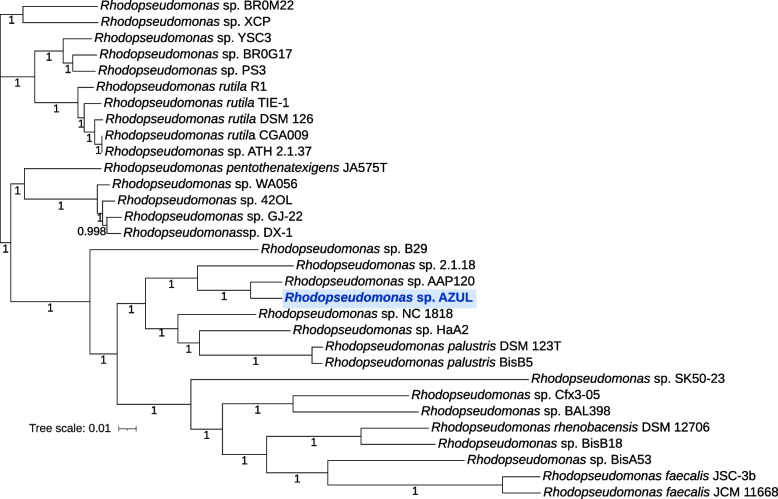
Unrooted tree of *Rhodopseudomonas* strains based on the core genome. Core genes obtained with Roary were concatenated, aligned and used to infer phylogenetic relationships with the neighbour joining method using FastTree, all part of the Roary pipeline. The tree scale in number of nucleotide substitutions per site is shown

With the purpose of determining which genes are common to the genus and which are specific to AZUL or to subgroups that include this strain, we did pangenomic analyses using the *Rhodopseudomonas* genome sequences of the strains in Table 1 that passed quality filtering by PATRIC (31 strains, Table S1). We compared the results of the softwares Roary and GET HOMOLOGUES (GH), and adjusted the data to the model developed by Tettelin et al*.* [33] and to the mixed binomial model [34]. The core genome (genes shared by all strains) obtained with the GH package was calculated using the clustering algorithms BDBH, OMCL and COGt, either independently or as a consensus between the three [29]. This produced core genomes of 1,055, 1,073, 1,041 and 987 gene clusters, respectively (Table 2). Roary [35] produced a core genome of 1,217 gene clusters (Table 2). The core genome predicted by Roary was used to build a phylogenetic tree of the relationships of the strains used in the pangenomic analysis. Figure 2 shows that, in agreement with the ANI analysis, AZUL is most closely related to *Rhodopseudomonas* sp. strain AAP120, and these two strains lie within larger clusters comprised of the *Rhodopseudomonas* sp. strains 2.1.18, NC 1818, B29 and HaA2 and the *Rhodopseudomonas palustris* strains DSM 123^ T^ and BisB5.

**Table 2:**
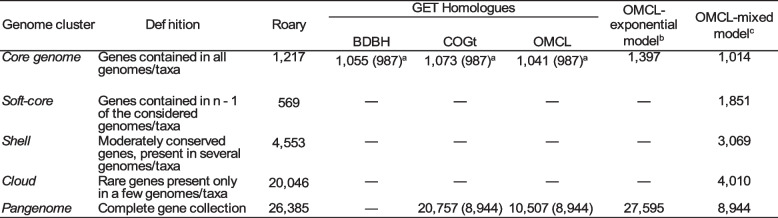
Pangenome characteristics of the genus *Rhodopseudomonas* based on different clustering algorithms

^a^The size of the core genome obtained from the consensus of the algorithms used by GH is shown between parentheses

^b^According to the model described in Tettelin et al.[33]

^c^According to the model of Snipen et al. [34]. In this model, the soft core includes the core, whereas it does not in Roary

**Fig. 3:**
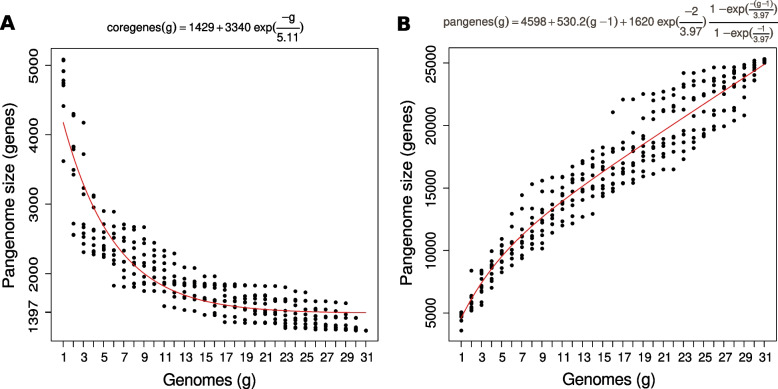
Pangenomic analysis of 31 *Rhodopseudomonas* strains using the exponential model. The number of gene clusters were plotted as a function of the number *n* of strains added sequentially. Black circles are the values obtained with the different orders in the addition of strains. The continuous red curves represent the least-squares fit of the data to an exponential function [33]. **A** Core genome plot. The core genome size with the addition of the 31st strain is 1,397. **B** Pangenome size trend. The equations are shown at the top of each plot

Figure 3 shows the adjustment of the core genome produced by OMCL-GH to the model used by Tettelin et al*.* [33]*,* which follows an equation of exponential decay. As expected, the number of genes shared by all strains decreased as more genomes were added to the analysis and reached a value of 1,397 gene clusters with the addition of the 31st strain (Fig. 3A and Table 2).

The size of the pangenome (the whole gene collection of the genus) was also estimated. Table 2 shows that Roary, COGt-GH and OMCL-GH predicted pangenomes of 26,385, 20,757 and 10,507 gene clusters, respectively. Figure 3B shows that adjustment of the OMCL-GH data to the model in Tettelin et al*.* [33] predicts a pangenome of 27,595 gene clusters when the 31st strain is added, similar to the results obtained with Roary.

The models described above have been criticized on the basis of the restrictions imposed by the classification of genes into only two categories (core genome and novel or specific genes). To overcome these restrictions, mixed models have been developed, such as one developed by Snipen et al*.* [34]. Table 2 shows that the core genome of the genus becomes much larger when the number of genes present in at least 30 strains are considered (*n-1*, soft core). In addition, it is noteworthy that a great proportion of genes comprised within the *Rhodopseudomonas* pangenome are either strain-specific or rare, the so called cloud genome.

The results presented above show that the precise characteristics of each algorithm markedly affects the estimation of the pangenome size. Despite these differences, our results evidence that the core genome comprises only a small proportion of the gene repertoire of *Rhodopseudomonas*, roughly between 5 and 12%. This was further evidenced by adjustment to the equations proposed by Tettelin et al*.* [33], since the addition of strains to the analysis increased the size of the total gene repertoire with a linear trend and reduced the size of the core genome. These results are expected, given the great metabolic adaptability of this genus. In this scenario, our results reinforce the idea that *Rhodopseudomonas* strains evolve from the core metabolic flexibility of the genus into the generation of distinct ecotypes highly adapted to specific microenvironments [2].

### Functional annotation of *Rhodopseudomonas* sp. AZUL

**Fig. 4:**
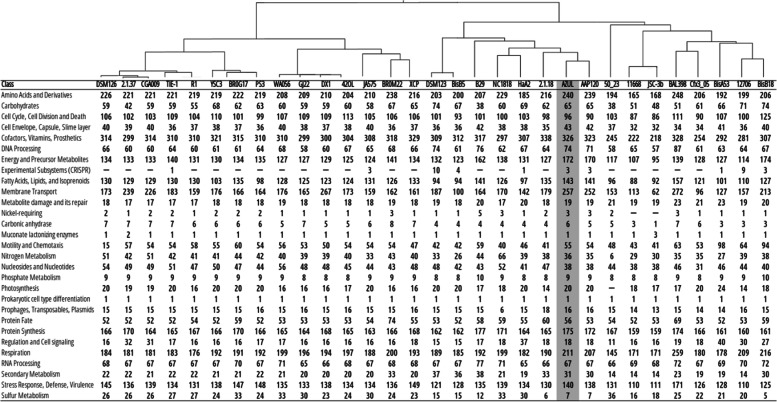
Gene count of main process classes in *Rhodopseudomonas* according to PATRIC. Comparisons between the 31 strains used for pangenomic analyses was done using PATRIC Comparative Pathways tool. AZUL is highlighted in gray. A similarity tree built using ANI values is shown

Functional annotation and clustering allowed determining which features of the AZUL genome are part of the basal capabilities of the *Rhodopseudomonas* genus (core, soft-core and shell genomes) and which are either strictly specific or shared with a few strains (cloud genome). For genome comparison, we selected the output of Roary, since it resulted adequate at discriminating paralogs in selected protein families, as determined in HMMER clustering analyses (data not shown). The identity cut-off for clustering was 70%. The proteins clustered separately through this method are not necessarily absent in other strains, but simply less than 70% identical to orthologous sequences from other strains. This percentage has been reported elsewhere as a suitable value for the determination of orthologs in interspecific analyses at the genus level [61]. All proteins that resulted specific or rare in the AZUL genome had nhmmer inter-cluster E-values that were at least four-fold higher than the corresponding intra-cluster E-values (data not shown). Table S1 shows Roary pangenomic clustering and annotation, including *Rhodopseudomonas* sp. AZUL predicted gene inventory. In addition, the total number of genes from different protein families, classes and subsystems were determined for the 31 strains using PATRIC (Fig. 4).

### Main predicted metabolic pathways of *Rhodopseudomonas* sp. AZUL (core, soft-core and shell genomes)

AZUL has the genes necessary for carrying out anoxygenic photosynthesis present in the other *Rhodopseudomonas* strains (Table S1). They include the light-harvesting complexes, electron carrier proteins, proteins involved in photo-phosphorylation and those linked to pigment biosynthesis.

Among the sequences associated with carbohydrate metabolism, we could identify genes related to glycolysis, gluconeogenesis, TCA and pentose-phosphate cycles. AZUL carbohydrate metabolism also features genes that participate in oligosaccharide and organic acid metabolism, fermentation and carbon dioxide fixation. One distinguishing characteristic of the *Rhodopseudomas* genus is the ability to degrade aromatic compounds, which, in AZUL, is represented by 30 main predicted genes (Table S1).

The subsystem of nitrogen metabolism includes proteins involved in nitrogen fixation (nitrogenases), ammonium assimilation and ammonification, nitrification and denitrification, cyanide hydrolysis and nitrosylative stress. Similarly to a few other strains, the genome of AZUL has genes for the three types of nitrogenases (iron, molybdenum and vanadium) (Table S1). Since they vary in their affinities for atmospheric N_2_ and in their catalytic efficacies [62], this would allow AZUL to fix nitrogen in the limited oligotrophic basin that constitutes its habitat [19]. *Rhodopseudomonas* strains have also shown the ability to form both monospecific biofilms [7] and mixed-species microbial mats [63], which could further limit the accessibility to nitrogen and other nutrients [64].

Similarly to other purple bacteria, the genome of AZUL has the genes to perform the four types of carbon and energy metabolism: photoautotrophy, photoheterotrophy, chemoautotrophy and chemoheterotrophy. AZUL has the genes for both form I and II of Ribulose-1,5-bisphosphate carboxylase/oxygenase (RuBiscCO), CbbM and CbbS, and a Rubisco-like protein (Rlp2), which enable this strain to carry out autotrophic growth. During this type of metabolism, hydrogen and thiosulfate could be used as electron donors in the reverse electron flow [3, 65]. Related to this, *Rhodopseudomas* strains have *hoxL* and *hoxK* genes*,* which code for hydrogenases. In addition, genes that code for several proteins involved in sulfate/thiosulfate uptake are shared between AZUL and the rest of the *Rhodopseudomas* strains, such as the import ATP-binding protein CysA, the thiosulfate sulfurtransferase GlpE and the thiosulfate-binding protein CysP*,* although some of these homologs seem to have diverged considerably between strains. A few of the strains, including AZUL, have the additional thiosulfate sulfurtransferase RhdA (Table S1).

Genes of carbon monooxide and formate dehydrogenases are also part of the collection of the genus. These proteins could contribute to generating reducing power through their oxidation. Other common dehydrogenases are related to ethanol and methanol metabolism, which, though not present in all strains, are part of the *Rhodopseudomas* repertoire (Table S1).

Within the subsystem of Respiration, 211 main sequences were identified in the genome sequence of AZUL, many within the core genome, including a great variety of oxidoreductases, cytochromes and proteins related to the biogenesis of these compounds. Oxidoreductase genes are either related to aerobic or anaerobic respiration. An example refers to the presence of a *dmsA* gene, which encodes a dimethyl sulfoxide/trimethylamine N-oxide reductase, an enzyme that catalyzes the reduction of dimethyl sulfoxide (DMSO) and trimethylamine N-oxide (TMAO) to dimethyl sulfide (DMS) and trimethylamine, respectively [66, 67] and can also use other sulfoxides and N-oxide compounds as terminal electron acceptors [68]. Another example is the *dsbB* gene, encoding a thiol-disulphur oxidoreductase involved in the reduction of the metalloid tellurium that is present in all the *Rhodopseudomonas* strains (Table S1). In *Rhodobacter capsulatus,* tellurium reduction due to the action of DsbB was proposed to act as an electric wire between the metalloid and the quinone pool [69], which suggests that this enzyme can be used for both detoxification and electron transport purposes.

The *Rhodopseudomonas* genus evidences an ability to synthesize multiple siderophore receptors. As an example, eleven sequences corresponding to the outer membrane receptor of the siderophore ferrichrome [70, 71] (five *fcuA* and six *fhuA* genes), as well as three sequences corresponding to the ferripyoverdine receptor FpvA [72] were identified in AZUL (Table S1).

The *Rhodopseudomonas* genus has a core ability to handle zinc limitation, although the gene sequences have diverged enough among strains to be located in different clusters (Table S1) Sequences of *zntB, znuC* and *yciC* were detected in all the strains including AZUL. The proteins encoded by these genes participate, in different ways, in Zn^2+^ uptake in conditions of metal limitation [73–75].

### Special predicted features of the *Rhodopseudomonas* sp. AZUL genome

Figure 4 shows that strains AZUL and AAP120 have remarkable similarities in the gene count of all the process classes analyzed by PATRIC. In general, these two strains are on the top end of gene count for most pathways, which could be, in part, due to the larger number of coding sequences present in their genomes. In general, this is not shared by other moderately related strains (those with ANI values above 85%).

#### Energy generation

Within the category of Energy and Precursor Metabolites Generation, it is remarkable in AZUL and AAP120 the presence of 4 genes that code for soluble methane monooxygenases (sMMO) (Table S1), present in only other 4 of the 31 strains analyzed (Table S1). This enzyme is a three-component non-heme iron oxygenase that catalyzes the initial step of the methane oxidation pathway, the conversion of methane to methanol. SMMOs can co-oxidize a very wide range of substrates together with methane, including alkanes, alkenes, alcohols, ethers, alicyclics, aromatics and chlorinated organic compounds such as the pollutant trichloroethene [76].

#### Urea and nitrogen metabolism

Within the category of Amino Acids and Derivatives, AZUL and AAP120 have a considerably larger number of urea amidolyase and urease genes (Table S1) than the rest of the *Rhodopseudomonas* strains (Table S1). Both enzymatic complexes participate in the utilization of urea as a nitrogen source by its sequential transformation to ammonium [77, 78]. A larger number of genes might allow more efficient nitrogen utilization under a wider array of conditions. As mentioned for nitrogenases, this would prove very useful in an oligotrophic environment with changing levels of nitrogen from various origins.

#### Membrane transport

The category of Membrane Transport is represented by 257 genes in AZUL and 252 genes in AAP120, only surpassed by *Rhodopseudomonas* sp. strain DX1 (267 genes) (Fig. 4). A closer inspection shows that, despite the precise gene count, different strains have different gene distribution within the subsystems of this class. Remarkable to AZUL and AAP120 are the number of genes involved in metal homeostasis, such as proteins that participate in copper and magnesium transport and multi-subunit cation antiporters (Table S1). In view of this, we did a semi-quantitative physicochemical analysis of the water sample where the strain was originally isolated from. Table 3 shows that metals and metalloids such as copper, zinc, chromium and arsenic are present in the water. Due to the fluctuating nature of the habi﻿tat of this strain, these results evidence, at the very least, that AZUL is periodically exposed to these metals.

**Table 3:** Elements present in the Laguna Azul water sample as determined by Inductively Coupled Plasma Mass Spectrometry (ICP-MS)

Element	Parts per billion (ppb)
B	10,640.76
Si	6,378.11
Li	2,948.40
P	1,240.92
Br	627.62
I	120.90
Fe	94.25
As	58.23
Rb	20.57
Cr	18.05
Xe	15.57
V	12.17
Ti	11.75
Se	6.20
Sr	6.10
Mo	3.56
U	2.29
Zn	2.04
Cu	2.00
Sc	1.74
Al	1.55
Ba	1.17

#### Copper homeostasis

Within this category are Copper-translocating P-type ATPases CopA, CopB and ActP, as well as the copper resistance proteins CopD and C and the repressor CsoR of the *copZA* operon. While the genomes of most *Rhodopseudomonas* strains have at least 1 *copA* gene homolog, a *copB* ortholog was only detected in the genome of AZUL. Furthermore, *actP* homologs were identified in strains AZUL, BisB5, BAL398 and DSM123 (Table S1). Both *csoR* orthologs, as well as sequences of its homolog *ricR* (one *ricR1* and one *ricR2*) were also found in AZUL.

Table 4 summarizes the number of genes related to metal homeostasis detected in the AZUL genome, as well information regarding their protein families and general mechanisms. In addition to translocating P-type ATPases, copper (and silver) resistance in bacteria is exerted via the resistance–nodulation–cell division (RND) metal efflux superfamily [79], which includes the partner proteins named membrane fusion protein (MFP) and outer membrane factors (OMF) [80]. These genes are part of the operon *cusCFBA* [81]. Although the *cus* operon is widespread in *Rhodopseudomonas*, AZUL has a larger number of genes compared to the rest of the strains, precisely 11 (Table 4).

**Table 4:**
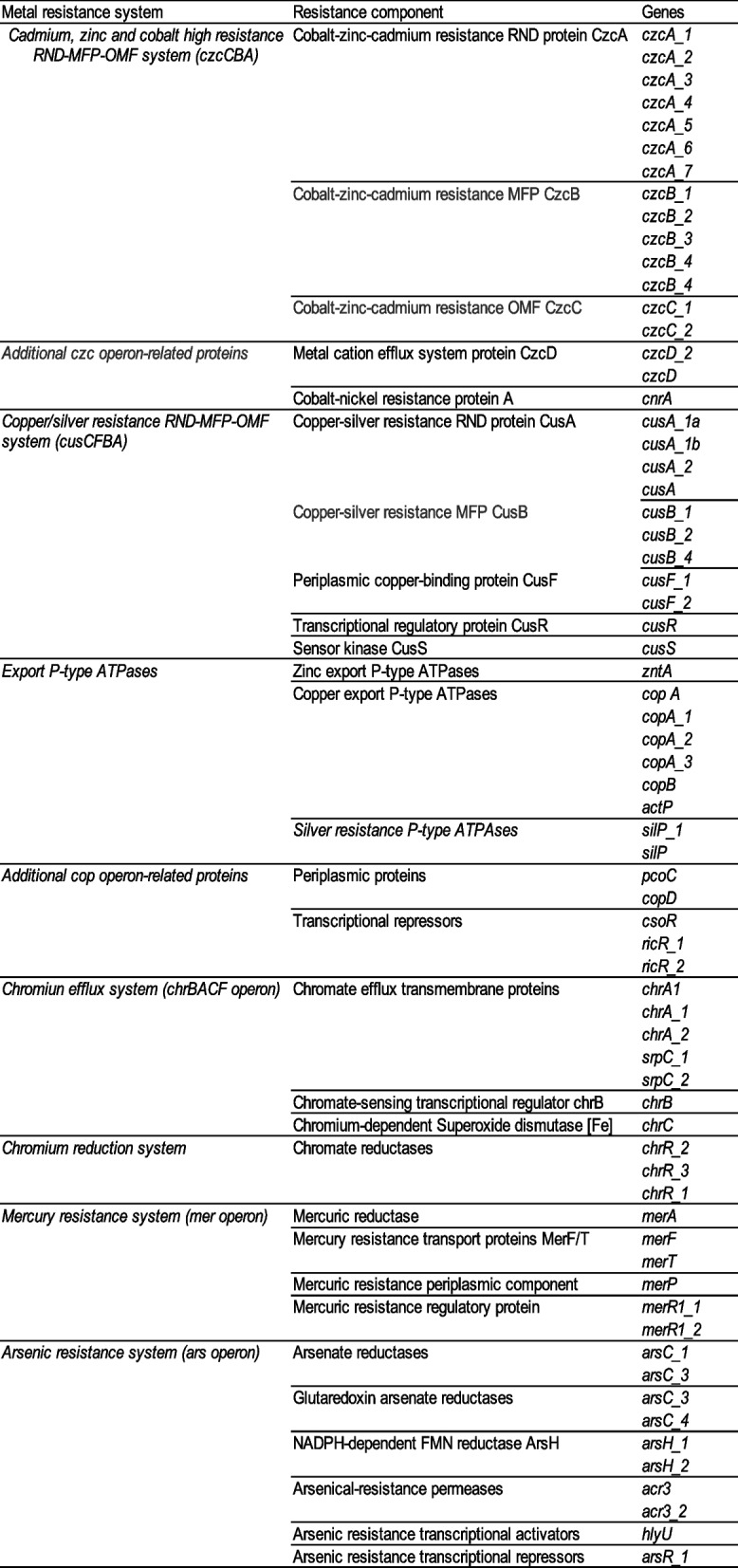
Main genes involved in metal resistance in *Rhodopseudomonas sp. *AZUL

**Fig. 5:**
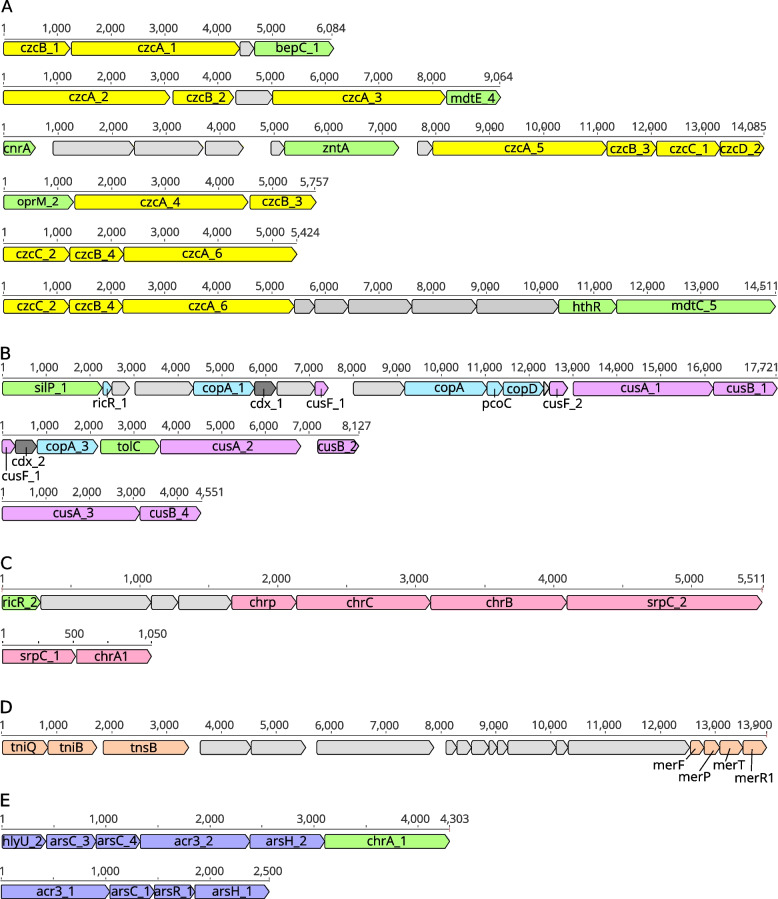
Schematic view of the main operons involved in metal resistance in the AZUL genome. **A** Genes of the *czc* system for resistance to cadmium, cobalt and zinc (yellow boxes). **B** Copper resistance *cop* (blue boxes) and *cus* (purple boxes), cupredoxin genes (*cdx*_*1*_ and *cdx-2,* dark gray boxes), *silP* and *tolC* (green). **C** Chr chromate efflux genes c*hrA, chrB*, *chrC*, *srpC* and *chrp* (pink boxes). **D** Mercury resistance genes *merP, merF, merT* and *merR1* genes, located in the vicinity of *tniQ, tniB* and *tnsB* genes, which participate in transposition (all orange boxes). **E** The main regulatory units of the AZUL arsenic resistance system (blue boxes) contain *arsC, acr3, arsH* and regulatory *arsR* and *hlyU* genes. In all the operons, box arrowheads show the orientation of each coding region. Light gray boxes represent unrelated or hypothetical coding regions and green boxes represent genes for resistance to other stressors. The numbered black lines indicate the size of each operon in base pairs. The scale is maintained for each resistance system, but different systems have different scales, in bp. For details about the function of each gene, please refer to Table 4

Figure 5 shows the organization of metal homeostasis genes within the *Rhodopseudomonas* sp. strain AZUL genome. The *cus* system in AZUL is represented by three main operons (Fig. 4B), in two cases intercalated with *cop* genes, forming larger copper-resistance units that also contain cupredoxin genes (*cdx*). Cupredoxins function as electron shuttles between proteins, which opens the possibility that AZUL uses copper for bioenergetic processes, as reported for other organisms [82]. These copper resistance units are located in the proximity of other genes related to resistance, such as the gene for a multidrug outer membrane efflux protein, *tolC* [83].

Several sequences that code for extracellular proteins involved in copper sequestration were identified. For instance, two homologous genes of the mycobacterial multicopper oxidase, *mmcO,* were detected in the genome of AZUL and three in the genome of BAL398, as opposed to single sequences in the rest of the strains (Table S1). MmcO possibly acts by oxidation of toxic Cu(I) in the periplasm [84]. In addition, single copies of the *pcoC* gene were detected in the genome of AZUL and several other strains (Table S1). PcoC is a periplasmic protein that binds excess copper ions and increases the level of resistance above that conferred by the *copA* operon alone [85]. The *pcoC* gene is part of one of the two *cop* operons present in AZUL, together with a single *copD* gene, which encodes a copper resistance protein of unclear function [86] (Fig. 4B).

CopA and CopB detoxify copper under aerobic conditions in many bacteria [87], whereas the *cus* operon is most frequently activated under anaerobic conditions [81]. In addition, actP ATPase exports copper at low pH [88]. Thus, the genome sequence of AZUL suggests that this strain is well appointed for copper export under varying environmental conditions, as well as for neutralizing toxic copper ions in the periplasm. The presence of the *ycnJ* and *ricR* genes, which code for transcriptional repressors [89, 90] evidences that AZUL can also respond to copper limitation. The location of an extra copy of the silver resistance gene *silP* adjacent to the *cus* operon suggests that, similar to other bacteria [91], the *cus* operon might be used for both silver and copper detoxification in AZUL.

#### Czc resistance operons

Another RND protein within the Cation Transporters subsystem is CzcA, a cation-proton antiporter that transports excess Cd^2 +^, Zn^2 +^ , and Co^2 +^ whose gene is located within an operon that contains genes for the MFP CzcB and the OMF protein CzcC [92]. Fourteen *czcA*, *czcB* and *czcC* genes were identified in the genome of AZUL (Table 4), most of them shared with other strains, although a few additional homologs were identified. The Czc system of AZUL is organized in at least six different operons, in some cases nearby other genes involved in efflux and resistance, such as those of the lead, cadmium, zinc and mercury-transporting ATPase ZntA, the cobalt–nickel resistance protein CnrA, the outer membrane efflux protein OprM and the multidrug efflux proteins MdtC, MdtE and BepC (Fig. 4A). One of these operons includes a gene for the cation diffusion facilitators (CDF) CzcD*,* which, in AZUL, is represented by single *czcD1* and *czcD2* gene sequences. CzcD was first described as a regulator of expression of the CzcCBA operon described above, but this protein is also able to mediate a small degree of Zn^2+^/Co^2+^/Cd^2+^ resistance in the absence of the high resistance CzcCBA system [93], which implies that the strains expressing this protein have the ability to activate low-level metal efflux.

#### Chromium resistance

Additional genes that participate in metal homeostasis/resistance were identified within the Stress Response, Defense and Virulence gene class. Many of them are present in most strains, albeit in different numbers. For instance, the genomes of AZUL and AAP120 feature a larger number of genes involved in chromium resistance than the rest of the strains (7 genes each, Table S1). These genes belong to the CHR family, composed of transmembrane proteins that act as chromate efflux pumps driven by a chemiosmotic gradient [94]. Three copies of both *chrA1* and *chrA2* genes were identified in the genome of AZUL, as well as two copies of the *chrA* homolog *srpC* and one copy of the transcriptional regulator gene *chrB* [95]. Some of the genes involved in chromate efflux are organized in AZUL within two main operons (Fig. 5C), while the rest of the copies are located in other parts of the genome.

Chromate reductase genes *chr1* and *chr2* were identified in the genomes of all the *Rhodopseudomonas* strains, whereas *chr3* homologs were only found in AZUL and a few other strains. In addition, single gene copies of the chromium-activated superoxide dismutase SodM-like protein ChrC/F were identified in AZUL and AAP120. Chromate reduction confers resistance to this metal in conjunction with chromate transport [96].

#### Mercury resistance

A cluster of sequences homologous to genes of the *mer* operon were identified in AZUL, namely one *merA*, two *merR*, two *merT/F* and one *merP*. MerA is mercury reductase, which catalyzes the reduction of Hg(II) to volatile Hg(0), while the rest of the *mer* genes encode proteins involved in regulation (MerR), cytoplasmic transport (MerT/F) and periplasmic binding (MerP) [97]. *Mer* sequences were found in other 8 of the *Rhodopseudomonas* strains, although only one *merR* and no *merT* were identified in any of them (Table S1). Interestingly, close to the AZUL *mer* operon, we identified *tniQ, tniB* and *tnsB* genes, which participate in transposition of the *mer* operon (Fig. 4D) [98], suggesting that this could have been the mechanism for acquisition of these genes in AZUL.

#### Arsenic resistance

We detected sequences homologous to genes from the *ars1 or ars2* operons, which confer resistance to arsenite and arsenate in a number of species [99, 100]. Specifically, *arsC1* and *arsC2* (arsenate reductases)*, acr3* (bile/arsenite/riboflavin-type permease), and *arsR* (transcriptional repressor) were identified in all the *Rhodopseudomonas* strains (Table S1). Additional sequences for *arsH1* and *arsH2* were only found in AZUL, with only one ortholog present in a few of the *Rhodopseudomonas* strains. ArsH proteins are organoarsenical oxidases that detoxify trivalent methylated and aromatic arsenicals by oxidation to the pentavalent species [101]. Contrary to CGA009 [102], we did not find evidence of an arsenite S-adenosylmethionine methyltransferase gene (*arsM*) in the genome of AZUL, suggesting that the pathway for As (III) methylation and subsequent volatilization through this protein is not among the mechanisms of resistance to arsenic in this strain. The *ars* genes are organized in two main operons in AZUL, one of them upstream of a chromate transporter gene *chrA* (Fig. 4E).

#### Multidrug resistance

The genomes of *Rhodopseudomonas* sp. AZUL and AAP120 feature many genes that take part in efflux systems for multidrug resistance, represented in AZUL by 94 detected genes, among the largest number detected in the 31 *Rhodopeudomonas* strains (Table S1). Multidrug efflux pumps export a wide variety of compounds in bacteria [103]. Interestingly, several multidrug genes within the AZUL genome were identified contiguous to genes of metal resistance (Fig. 4).

The appearance of multidrug resistance in conjunction with resistance to metals within the same genome might not be casual. A study conducted in Laguna Azul showed a correlation between multi-resistance to UV radiation, arsenic and antibiotics, despite the fact that selective pressure for antibiotic resistance is not expected in that habitat [12]. The authors proposed that, under the extreme UV radiation that the HAALs are exposed to, bacteria have increased mutation rates in which spontaneous resistance to antibiotics can emerge. Indeed, our results show that, in AZUL, multidrug genes are often located adjacent to metal resistance operons, which could have important implications related to their origin and/or regulation.

#### Protein and nucleoprotein secretion system, Type IV

Within the Membrane Transport class, AZUL, AAP120 and BAL398 have several genes involved in Vir-like type 4 secretion (Table S1). The type IV (T4SS) is one of several types of secretion systems that microorganisms use for the transport of macromolecules such as proteins and DNA across the cell envelope [104]. It is the most versatile family of secretion, mediating transport of monomeric proteins as well as multi-subunit protein toxins and nucleoprotein complexes. In the case of DNA transfer, it is known to greatly increase genomic plasticity, helping microorganisms to adapt to changes in their environment [104].

#### Metal uptake

Many genes that take part in metal uptake are common among all or most of the *Rhodopseudomonas* strains. One exception is the *mgtB* gene, which encodes a P-type ATPase involved in Mg^2+^ intake under limiting conditions [105] that was only detected in AZUL. In addition, two homologs of the *hoxN* gene, involved in high-affinity nickel uptake [106] are present in AZUL, while only one homolog, or none, were found in the rest of the strains (Table S1).

#### Response to UV

The sequences related to tolerance to UV are, in general, common between AZUL and the rest of the *Rhodopseudomonas* strains. Nevertheless, there are a few exceptions. For instance, single homologs encoding RuvA and RuvB, the subunits of an ATP-dependent DNA helicase that participates in recombinational repair of UV damage [107], were identified in all the *Rhodopseudomonas* strains, while an extra gene for a RuvB subunit was only found in the genome sequence of AZUL. As another example, homologs of the deoxyribodipyrimidine photolyase gene *phrA,* involved in photorepair [108], were identified in all the strains analyzed, whereas its counterpart, *phrB*, was only identified in AZUL and 2.1.18.

## Conclusions

In this work, we have presented the draft genome sequence of *Rhodopseudomonas sp.* AZUL, a strain isolated from an endorheic basin in the Argentinean Andean region. In addition, we have done comparative genomic analyses that included most of the *Rhodopseudomona*s strains with available sequenced genomes. This has led us to conclude that the *Rhodopseudomonas* genus has an open pangenome, as the addition of strains to the analysis increased the size of the total gene repertoire with a linear trend. These results are expected given the great metabolic adaptability of this genus. In this scenario, our results reinforce the idea that *Rhodopseudomonas* strains evolve from the basal metabolic flexibility of the genus into the generation of ecotypes greatly adapted to their specific niches [2]. Our genomic analysis has shown that, in addition to the metabolic complexity of the genus, AZUL has numerous mechanisms for both the uptake and export of minerals and other chemicals, as well as for chemical detoxification. Being able to accomplish the four types of carbon and energy metabolism, it is likely that AZUL has evolved to not only tolerate but also use some of these compounds as electron carriers.

The genome of AZUL has also several transport genes for DNA and nucleoprotein uptake, which could confer remarkable genomic plasticity that surely helps this strain adapt to changes in its surroundings. This is ideal considering that the natural habitat of AZUL experiences drastic changes in environmental parameters and seasonal contraction and expansion of the water levels that modify the amount of minerals and other compounds from geochemical and volcanic origin [17, 109]. In addition to a remarkable adaptation to a hostile environment, the genome of AZUL makes us envision promising industrial applications of this microorganism. Future experimentation will interrogate its capacity to bioremediate both metals and organic compounds.

## Supplementary Information

**Additional file 1.** Report for *R. palustris *AZUL genome sequencing and assembly. Project type: WGS.

**Additional file 2: (Table S1):Table S1-1.** Results of genome quality analysis of the 39 Rhodopseudomonas strains used in this work, obtained using the Genome Annotation service at PATRIC. **Table S1-2.** Full clustering analysis of the 31 strains of Rhodopseudomonas with Roary, using 70% identity cut-off and the ‘do not split paralogs’ option. **Table S1-3.** Gene annotation of the Rhodopseudomonas genome according to Prokka and Roary (split paralogs option). **Table S1-4.** Gene count of selected subsystems from the PATRIC comparative pathway output in the 31 Rhodopseudomonas strains.

## Data Availability

This whole-genome shotgun project is available at the RAST server (https://rast.nmpdr.org/) and also at NCBI GenBank, with the assembly accession GCA_024330085.1.
