# Supplementary material for: The draft genome of Andean Rhodopseudomonas sp. strain AZUL predicts genome plasticity and adaptation to chemical homeostasis
Source: BMC Microbiol. 2022 Dec 9;22:297. doi: 10.1186/s12866-022-02685-w (PMC9733117; doi:10.1186/s12866-022-02685-w)
Supplement: Supplementary file 1 — Additional file 1. Report for R. palustris AZUL genome sequencing and assembly. Project type: WGS. [file 12866_2022_2685_MOESM1_ESM.pdf]

Report for *R. palustris* AZUL genome sequencing and assembly. Proyect type: WGS

Preparation of the TruSeq DNA libraries (Illumina)

Two micrograms of purified DNA were resuspended in 50 µL of TE buffer. DNA fragmentation by nebulization, repair and end-adenylation, adapter ligation, gel purification and enrichment (amplification) were done according to the protocol described in TruSeq® DNA Sample Preparation Guide, Illumina.

Quality control of DNA libraries

One microgram of the DNA library was run in a 2100 Bioanalyzer (Agilent Technologies) using the High Sensitivity DNA kit. The library was composed of double-stranded DNA fragments of sizes ranging 300-500 bp. The libraries were of enough quality to proceed with the sequencing.

Library quantification

The library was quantified by qPCR (Light Cycler 480 Roche) using the Kapa Library Quantification kit and normalized to 2 nM. One equimolar pool of the libraries from the same sequencing lane was prepared by mixing 10 µl of each. The pool was used for the generation of clusters in a single lane of the sequencing cell.

Statistical analysis of the HiSeq1500 sequencing run

Paired-end (PE) sequencing was done (2 x 100 bp). Statistical data of the rapid run (2 lanes) were as follows:

Level	Cycles	Yield (Total)	Projected (Total)	Intensity	% Intensity	% >= 0.30
Read 1	101	31.7 G	31.7 G	5142	76.1	92.4
Read 2 (I)	7	1.9 G	1.9 G	5033	0	96.1
Read 3	101	31.7 G	31.7 G	4943	77.2	88.9
**Total**	**209**	**65.2 G**	**65.2 G**	**5039**	**76.7**	**90.8**

Cluster PF (%)	Phas/Prephas (%)	Reads (M)	Reads PF (M)	% > 0.30	Yield	Intensity Cycle 1	intensity Cycle 2
94.45 +/- 0.83	0.094/0.096	166.10	156.76	92.1	15.7 G	5114 +/- 153	75.5 +/- 2.2
94.21 +/- 0.82	0.095/0.103	169.69	159.75	92.6	16.0 G	5170 +/- 159	76.6 +/- 1.9
94.45 +/- 0.83	0.000/0.000	166.10	156.76	96.2	940.6 M	5023 +/- 166	0.0 +/- 0.0
94.21 +/- 0.82	0.000/0.000	169.69	159.75	96.0	958.5 M	5043 +/- 183	0.0 +/- 0.0
94.45 +/- 0.83	0.108/0.119	166.10	156.76	88.9	15.7 G	4937 +/- 185	77.2 +/- 2.3
94.21 +/- 0.82	0.111/0.125	169.69	159.75	88.9	16.0 G	4950 +/- 175	77.2 +/- 1.3

Cluster density was 600 K/mm^2, better than the average value expected for this technology. The data had good quality parameters, with a 90.8% of all the lectures with a Qscore >Q30 (error rate <0.001, probability of incorrect base call < 1 in 1,000 bases, base call accuracy > 99.9 %). After separation of the reads the results were the following:

Total Reads	PF Reads	% Reads identified (PF)	CV
22,300,084	22,108,303	99.14	0.16

<u>Assembly, annotation and genome quality analysis</u>

Genome assembly starting from paired-end reads was done using the a5pipeline v20140113. Quality of the assembly (completeness, contamination and annotation self-consistency) were estimated using the genome-quality analysis service 'EvalG', which runs within the Genome Annotation service at PATRIC. Preliminary genome annotation was done using the RAST toolkit. The genome of *R. palustris* AZUL was estimated to be 100% complete, as shows the following table:

Genome Report (Genome Annotation tool in PATRIC)	
Genome ID	1076.72
Genome Name	Rhodopseudomonas palustris AZUL
Reference Genomes	258594.8
Coarse consistency (%)	99
Fine consistency (%)	95.5
Completeness (%)	100
Contamination (%)	2
Evaluation Group	R100 (Bradyrhizobium sp. ORS 278)

Contig count	120
DNA size (bp)	6050441
Contigs N50 (bp)	272881
Contigs L50	6
Overpresent Roles	58
Underpresent Roles	5
Predicted Roles	1479
Completeness Roles	435
Total Distinct Roles	3079
Protein-Encoding Genes with Functional Assignment	3520
Protein-Encoding Genes without Functional Assignment	2238
% Protein-Encoding Feature Coverage	95.17
% Features that are Hypothetical	38.87
% Features that are in Local Protein Families	88.31

The *R. palustris* AZUL genome sequence is available at the RAST server (https://rast.nmpdr.org/), using Login: guest and Password: guest and also at NCBI GenBank, with the assembly accession is GCA_024330085.1 (https://www.ncbi.nlm.nih.gov/assembly/GCA_024330085.1).
